# Supplementary material for: Evaluation of Intraligamentous and Intraosseous Computer-Controlled Anesthetic Delivery Systems in Pediatric Dentistry: A Randomized Controlled Trial
Source: Children (Basel). 2022 Dec 30;10(1):79. doi: 10.3390/children10010079 (PMC9856416; doi:10.3390/children10010079)
Supplement: Supplementary file 1 [file children-10-00079-s001.zip › children-2120709-supplementary.pdf]

Table S1. Computer delivery system-intraligamentary anesthesia versus inferior alveolar nerve block with conventional syringe. Strength of the relationship in the proposed model between exposure to a considered risk factor (covariates of the first column) and the outcome variables (first row).

	**Pain due to the anesthesia injection[a]**	**Physical reaction during the anesthesia injection[b]**	**Anesthetic reinforcement**	**Pain during the therapeutic procedure[a]**
**Sex**	ns	ns	ns	OR=3.800<br>CI=1.056–13.740<br>p=0.040
**Age**	ns	ns	OR=3.954<br>CI=0.911–17.104<br>p<0.001	ns
**Dental procedure**	ns	ns	ns	ns
**Anesthesia system**	OR=6.410<br>CI=1.723–23.817<br>p=0.010	EC=1.125<br>SE=0.222<br>p<0.001	ns	ns

Table S1. (continue).

	**Overall behavior during the visit[c,d]**	**Postoperative morbidity**	**Type of postoperative complication[e]**
**Sex**	ns	ns	ns
**Age**	ns	ns	ns
**Dental procedure**	ns	[f] ns<br><br>[g] OR=11.580<br>CI=1.421–94.473<br>p=0.02<br><br>[h] OR=14.490<br>CI=1.301–161.954<br>p=0.033	ns
**Anesthesia system**	ns	OR=14.661<br>CI=2.697–79.903<br>p<0.001	OR=3.980<br>CI=0.991–15.957<br>p=0.021

[a] Recoded Wong-Baker score (binomial); [b] FLACC score (binomial); OR, Odds ratio; ns, not significant; CI, confidence interval; p, p-value; ; EC, estimated coefficient by the model; SE, standard error; [c] Frankl scale; [d] The data correspond to the extreme responses ("highly negative" versus "highly positive"); [e]The model is only reliable regarding "discomfort" versus "without complications" (not applicable with relation to "pain" or to "nibbling injuries"); [f]very deep versus deep fillings; [g] pulpotomies versus deep fillings ; [h] extractions versus deep fillings

Table S2. Computer delivery system-intraosseous anesthesia versus inferior alveolar nerve block with conventional syringe. Strength of the relationship in the proposed model between exposure to a considered risk factor (covariates) and the outcome variables (first row).

	Pain due to the anesthesia injection[a]	Physical reaction during the anesthesia injection[b]	Anesthetic reinforcement	Pain during the therapeutic procedure[a]
**Sex**	ns	ns	ns	ns
**Age**	ns	EC= -0.423<br>SE=0.154<br>p=0.008	ns	ns
**Dental procedure**	ns	ns	ns	ns
**Anesthesia system**	OR=0.107<br>CI=0.012–0.950<br>p=0.045	EC=-1.680<br>SE=0.639<br>p=0.011	ns	ns

Table S2. (continue).

	Overall behavior during the visit[c,d]	Postoperative morbidity	Type of postoperative complication[e]
**Sex**	ns	ns	ns
**Age**	ns	ns	ns
**Dental procedure**	ns	ns	ns
**Anesthesia system**	ns	OR=0.306<br>CI=0.094–0.992<br>p=0.048	OR=1.473<br>CI=0.658–1.894<br>p=0.043

[a] Recoded Wong-Baker score (binomial); [b] FLACC score (binomial); ns, not significant; EC, estimated coefficient by the model; SE, standard error; OR, Odds ratio; CI, confidence interval; p, p-value; ; [c] Frankl scale; [d] The data correspond to the extreme responses ("highly negative" versus "highly positive"); [e] The model is only reliable regarding "discomfort" versus "without complications" (not applicable with relation to "pain" or to "nibbling injuries")

Figure S1. Flow chart of the patient selection.

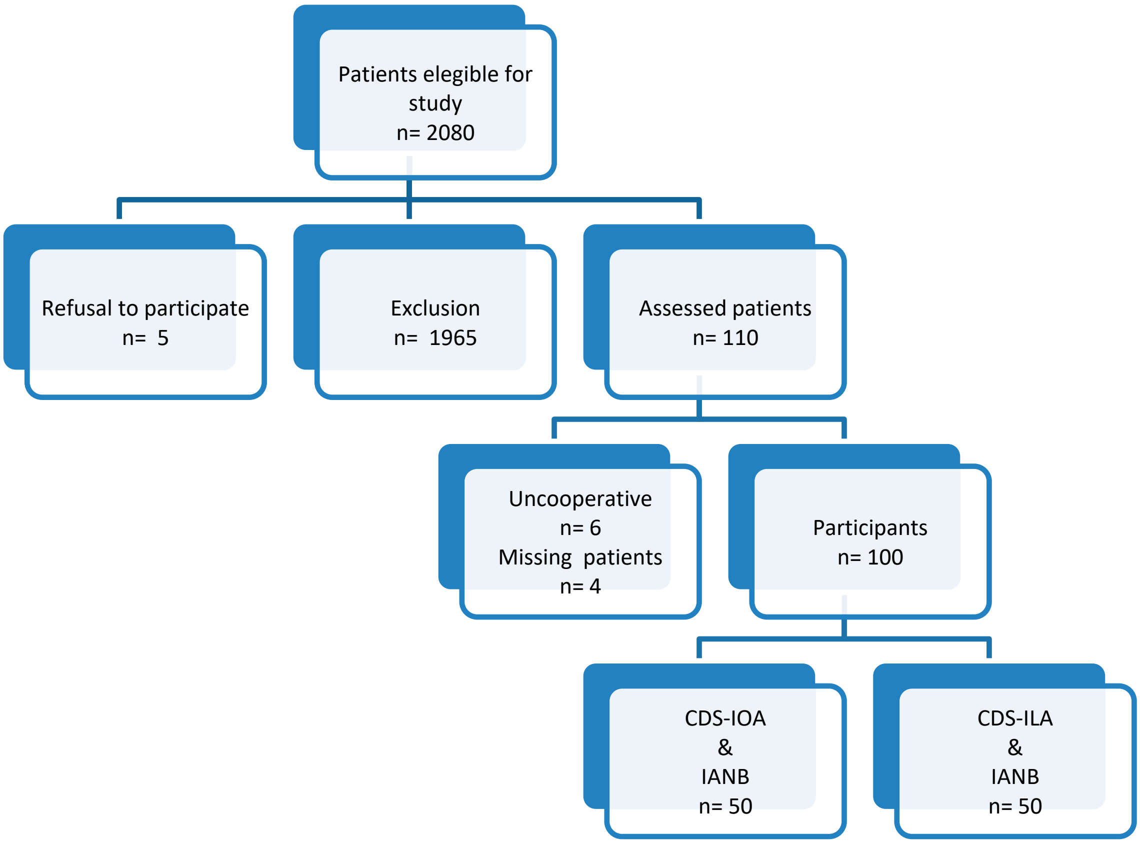

CDS-ILA, computer delivery system-intraligamentary anesthesia; CDS-IOA, computer delivery system-intraosseous anesthesia; IANB, inferior alveolar nerve block with conventional syringe.
